# Pulmonary arterial hypertension

**DOI:** 10.1186/1750-1172-8-97

**Published:** 2013-07-06

**Authors:** David Montani, Sven Günther, Peter Dorfmüller, Frédéric Perros, Barbara Girerd, Gilles Garcia, Xavier Jaïs, Laurent Savale, Elise Artaud-Macari, Laura C Price, Marc Humbert, Gérald Simonneau, Olivier Sitbon

**Affiliations:** 1 Univ. Paris-Sud, Faculté de Médecine, Kremlin-Bicêtre F-94270, France; 2 AP-HP, DHU TORINO, Centre de Référence de l’Hypertension Pulmonaire Sévère, Service de Pneumologie et Réanimation Respiratoire, Hôpital Bicêtre, Le Kremlin-Bicêtre F-94270, France; 3 INSERM U999, Labex LERMIT, Hypertension Artérielle Pulmonaire: Physiopathologie et Innovation Thérapeutique, Centre Chirurgical Marie Lannelongue, Le Plessis-Robinson F-92350, France; 4 Pulmonary Hypertension Service, Royal Brompton Hospital, London SW3 6NP, UK

## Abstract

Pulmonary arterial hypertension (PAH) is a chronic and progressive disease leading to right heart failure and ultimately death if untreated. The first classification of PH was proposed in 1973. In 2008, the fourth World Symposium on PH held in Dana Point (California, USA) revised previous classifications. Currently, PH is devided into five subgroups. Group 1 includes patients suffering from idiopathic or familial PAH with or without germline mutations. Patients with a diagnosis of PAH should systematically been screened regarding to underlying mutations of *BMPR2* gene (bone morphogenetic protein receptor type 2) or more rarely of *ACVRL1* (activine receptor-like kinase type 1), *ENG* (endogline) or *Smad8* genes. Pulmonary veno occusive disease and pulmonary capillary hemagiomatosis are individualized and designated as clinical group 1'. Group 2 'Pulmonary hypertension due to left heart diseases' is divided into three sub-groups: systolic dysfonction, diastolic dysfonction and valvular dysfonction. Group 3 'Pulmonary hypertension due to respiratory diseases' includes a heterogenous subgroup of respiratory diseases like PH due to pulmonary fibrosis, COPD, lung emphysema or interstitial lung disease for exemple. Group 4 includes chronic thromboembolic pulmonary hypertension without any distinction of proximal or distal forms. Group 5 regroup PH patients with unclear multifactorial mechanisms. Invasive hemodynamic assessment with right heart catheterization is requested to confirm the definite diagnosis of PH showing a resting mean pulmonary artery pressure (mPAP) of ≥ 25 mmHg and a normal pulmonary capillary wedge pressure (PCWP) of ≤ 15 mmHg. The assessment of PCWP may allow the distinction between pre-capillary and post-capillary PH (PCWP > 15 mmHg). Echocardiography is an important tool in the management of patients with underlying suspicion of PH. The European Society of Cardiology and the European Respiratory Society (ESC-ERS) guidelines specify its role, essentially in the screening proposing criteria for estimating the presence of PH mainly based on tricuspid regurgitation peak velocity and systolic artery pressure (sPAP). The therapy of PAH consists of non-specific drugs including oral anticoagulation and diuretics as well as PAH specific therapy. Diuretics are one of the most important treatment in the setting of PH because right heart failure leads to fluid retention, hepatic congestion, ascites and peripheral edema. Current recommendations propose oral anticoagulation aiming for targeting an International Normalized Ratio (INR) between 1.5-2.5. Target INR for patients displaying chronic thromboembolic PH is between 2–3. Better understanding in pathophysiological mechanisms of PH over the past quarter of a century has led to the development of medical therapeutics, even though no cure for PAH exists. Several specific therapeutic agents were developed for the medical management of PAH including prostanoids (epoprostenol, trepoprostenil, iloprost), endothelin receptor antagonists (bosentan, ambrisentan) and phosphodiesterase type 5 inhibitors (sildenafil, tadalafil). This review discusses the current state of art regarding to epidemiologic aspects of PH, diagnostic approaches and the current classification of PH. In addition, currently available specific PAH therapy is discussed as well as future treatments.

## Definition and classification

Pulmonary arterial hypertension (PAH) is defined by right-heart catheterization (RHC) showing precapillary pulmonary hypertension with a mean pulmonary artery pressure (mPAP) of >25 mmHg and a normal pulmonary artery wedge pressure (PCWP) of <15 mmHg [1,2]. The classification of pulmonary hypertension (PH) has gone through a series of changes since the first classification proposed in 1973 which designated only two categories, primary pulmonary hypertension or secondary PH, depending on the presence or absence of identifiable causes or risk factors [3,4]. In 1998, a second World Symposium on PH was held in Evian (France) and this classification attempted to create categories of PH that shared similar pathogenesis, clinical features and therapeutic options [5]. This classification allowed defining homogenous groups of patients to conduct clinical trials and to obtain approval for specific PAH therapies worldwide. In 2003, the third World Symposium on PH (Venice, Italy) did not propose major changes. However, the terms idiopathic PAH, familial PAH, and associated PAH were introduced. The other prominent change was to move pulmonary veno-occlusive disease (PVOD) and pulmonary capillary hemangiomatosis (PCH) from separate categories into a single subcategory of PAH.

In 2008, the fourth World Symposium on PH held in Dana Point (California, USA) and the consensus of an international group of experts was to revise previous classifications in order to accurately reflect published data, as well as to clarify some areas that were unclear. In 2013, the fifth World Symposium on PH held in Nice (France) and proposed only minor modifications, however, since the definite conclusions of this symposium were not yet published, we presented the Dana Point classification of PH (Table 1).

**Table 1:** Diagnostic classification of pulmonary hypertension

**1. Pulmonary arterial hypertension (PAH)**	1.1 Idiopathic
	1.2 Heritable
	1.3 Drugs and toxins induced
	1.4 Associated with (APAH):
	1.4.1 Connective tissue disease
	1.4.2 Infection with human immunodeficiency virus
	1.4.3 Portal hypertension
	1.4.4 Congenital heart disease
	1.4.5 Schistosomiasis
	1.4.6 Chronic haemolytic anaemia
	1.5 Persistent pulmonary hypertension of the newborn
**2. Pulmonary hypertension with left heart disease**	2.1 Systolic dysfunction
	2.2 Diastolic dysfunction
	2.3 Valvular disease
**3. Pulmonary hypertension due to lung diseases and/or hypoxia**	3.1 Chronic obstructive pulmonary disease
	3.2 Interstitial lung disease
	3.3 Other pulmonary diseases with mixed restrictive and obstructive pattern
	3.4 Sleep-disordered breathing
	3.5 Alveolar hypoventilation disorders
	3.6 Chronic exposure to high altitude
	3.7 Developmental abnormalities
**4. Chronic thromboembolic pulmonary hypertension**	
**5. PH with unclear and/or multifactorial mechanisms**	5.1 Haematological disorders: myeloproliferative disorders, splenectomy.
	5.2 Systemic disorders: sarcoidosis, pulmonary Langerhans cell histiocytosis, lymphangioleiomyomatosis, neurofibromatosis, vasculitis
	5.3 Metabolic disorders: glycogen storage disease, Gaucher disease, thyroid disorders
	5.4 Others: tumoral obstruction, fibrosing mediastinitis, chronic renal failure on dialysis

This classification was adapted from (Simonneau et al. [16]).

### Group 1: Pulmonary arterial hypertension

The nomenclature of the subgroups and associated conditions has evolved since the first classification, and additional modifications were added in the Dana Point classification.

#### *Group 1.1/1.2 Idiopathic and heritable PAH*

Idiopathic PAH describes a sporadic disease with neither a family history of PAH nor an identified risk factor. When PAH occurs in a familial context, germline mutations in the *bone morphogenetic protein receptor 2 (BMPR2)* gene, a member of the transforming growth factor beta (TGF- ß) signaling family, can be detected in about 70% of cases [6,7]. More rarely, mutations in *activin receptor like kinase type 1 (ACVRL1 or ALK1)* or *endoglin genes*, also coding for members of the TGF-ß signaling family, have been identified in patients with PAH, predominantly with coexistent hereditary hemorrhagic telangiectasia. Some authors suggested that mutations of genes encoding for Smads proteins (*Smad8*, *Smad1* and *Smad5*), which are other members of the TGF-ß signaling pathway, or mutations in *caveolin-1* gene may predispose to PAH [8-10].

*BMPR2* mutations have also been detected in 11–40% of apparently idiopathic cases with no family history [11,12]. Indeed, the distinction between idiopathic and familial PAH with *BMPR2* mutations is artificial, as all patients with a *BMPR2* mutation have heritable disease. In addition, *BMPR2* mutations were identified in only 70-80% families with PAH. Thus, it was decided to abandon the term “familial PAH” in favor of the term “heritable PAH”, including idiopathic PAH with germline mutations and familial cases with or without identified mutations [13,14].

#### *Group 1.3 Drug- and toxin-induced PAH*

A number of risk factors for the development of PAH have been individualized in the last European Respiratory Society/ European Society of Cardiology (ERS/ESC) conjoint guidelines of PH [15] (Table 2).

**Table 2:** Updated risk level of drugs and toxins known to be associated with PAH

1. Definite	
	Aminorex
	Fenfluramine
	Dexfenfluramine
	Toxic rapeseed oil
	Benfluorex
2. Likely	
	Amphetamines
	L-tryptophan
	Metamphetamines
	Dasatinib
3. Possible	
	Cocaine
	Phenylpropanolamine
	St John’s Wort
	Chemotherapeutic agents
	Selective serotonin reuptake inhibitors
	Pergolide
4. Unlikely	
	Oral contraceptives
	Estrogen therapy
	Cigarette smoking

*This table was adapted from Galiè et al. [15].

Aminorex, fenfluramine derivatives and toxic rapeseed oil represent the only identified “definite” risk factors for PAH [5,16]. Souza et al. have demonstrated that this subgroup of PAH shares clinical, functional, hemodynamic, and genetic features with idiopathic PAH, suggesting that fenfluramine exposure represents a potential trigger for PAH without influencing its clinical course [17].

Two prospective epidemiologic investigations, the SNAP (Surveillance of North American Pulmonary Hypertension) and the SOPHIA (Surveillance of Pulmonary Hypertension in America) study, were conducted in the USA [18,19]. These investigations included retrospectively 559 and 1335 patients with PH, respectively, and confirmed the previously described association between idiopathic PAH and the use of fenfluramine. In the SNAP study, the odds ratio of developing PH was 7.5 for the use of fenfluramine more than six months of treatment [18].

The agent benfluorex is structurally and pharmacological related to fenfluramine and may be also considered as an anorectic agent. Frachon and co-workers [20] showed a significantly higher prevalence of unexplained valvular heart disease in patients taking benfluorex compared to controls. In addition, Savale and co-workers demonstrated recently that exposure to benfluorex is suggested to be a trigger in the development of PAH [21]. Benfluorex was withdrawn from the market in 2009.

The SOPHIA study examined intake of a variety of nonselective monoamine reuptake inhibitors, selective serotonin reuptake inhibitors, antidepressants and anxiolytics. No increased risk of developing PAH was observed [19].

Amphetamine use represents a “likely” risk factor, although they are frequently used in combination with fenfluramine. A recent retrospective study suggested a relationship with the use of methamphetamine (inhaled, smoked, oral, or intravenous) and the occurrence of PAH [22]. Methamphetamine use is now considered a “very likely” risk factor for the development of PAH.

Recently published data from the French registry of pulmonary hypertension suggested that dasatinib, a tyrosine kinsase inhibitor (TKI), may induce precapillary PAH [23]. Several cases of precapillary PH in chronic myelogenous leukemia patients treated with dasatinib have been reported.

#### *Group 1.4.1 PAH associated with connective tissue diseases*

PAH associated with connective tissue diseases (CTD) represents an important clinical subgroup, in which systemic sclerosis represents the major cause of CTD associated PAH. The prevalence of PAH has been well established only for systemic sclerosis (SSc). Prospective studies using echocardiography as a screening method and RHC for confirmation found a prevalence of PAH between 7–12% [24,25]. PH due to lung fibrosis [26], diastolic left heart dysfunction [27] and primary cardiac involvement [28] are also frequent in the setting of pulmonary hypertension in these patients, emphasizing the importance of a systemic evaluation with RHC to accurately classify the underlying mechanism of PH.

#### *Group 1.4.2 HIV infection*

PAH is a rare complication of HIV infection [29,30]. HIV-associated PAH has clinical, hemodynamic, and histologic characteristics broadly similar to those seen in idiopathic PAH. Epidemiologic data in the early 1990s, a time when therapy with highly active antiretroviral therapy was not yet available, indicated a prevalence of 0.5% [31]. The prevalence of HIV-associated PAH was evaluated more recently and showed a stable prevalence of 0.46% [32].

#### *Group 1.4.3 Porto-pulmonary hypertension*

Porto-pulmonary hypertension (POPH) is defined by the development of PAH associated with increased pressure in the portal circulation [33,34]. Prospective hemodynamic studies have shown that 2-6% of patients with portal hypertension had PH [35,36]. However, RHC is mandatory for the diagnosis of portal PH, as several mechanisms may increase pulmonary artery pressure in the setting of advanced liver disease: hyperdynamic circulatory state with high cardiac output, fluid overload and diastolic dysfunction. Pulmonary vascular resistance (PVR) is usually normal in these cases.

#### *Group 1.4.4 Congenital heart diseases*

A significant proportion of patients with congenital heart disease (CHD), in particular those with systemic-to-pulmonary shunts, will develop PAH if left untreated. Eisenmenger's syndrome is defined as CHD with an initial large systemic-to-pulmonary shunt that induces progressive pulmonary vascular disease and PAH, with resultant reversal of the shunt and central cyanosis [37,38]. It represents the most advanced form of PAH associated with CHD. It has been reported that a large proportion of patients with CHD develop some degree of PAH [39-41]. The prevalence of PAH associated with congenital systemic-to-pulmonary shunts in Europe and North America has been estimated between 1.6 and 12.5 cases per million adults, with 25-50% of this population affected by Eisenmenger’s syndrome.

#### *Group 1.4.5 Schistosomiasis*

In the Dana Point classification, PH associated with schistosomiasis was included in Group 1. Recently, it has been demonstrated that PH associated with schistosomiasis may have a similar clinical presentation and histological findings as idiopathic PAH [42,43]. The mechanism of PAH in patients with schistosomiasis is probably multifactorial including portal PH, a frequent complication of this disease [44] and local vascular inflammation, whereas mechanical obstruction by schistosoma eggs seems to play a minor role. More than 200 million people are infected and 4–8% of them will develop hepatosplenic disease. Then, PAH associated with schistosomiasis represents a frequent form of PAH in countries where the infection is endemic. Data from a recent study based on invasive hemodynamics evidenced the prevalence of PAH in patients with hepatosplenic disease of 4.6%; also important was the prevalence of post-capillary hypertension (3%) reinforcing the need of invasive hemodynamics for the specific diagnosis of PAH in schistosomiasis [45].

#### *Group 1.4.6 Chronic hemolytic anemia*

The chronic hemolytic anemias represent a subcategory of PAH. There has been increasing evidence that PAH is a complication of chronic hereditary and acquired hemolytic anemias, including sickle cell disease [46,47], thalassemia [48], hereditary spherocytosis [49], stomatocytosis [50], and microangiopathic hemolytic anemia [51].

PH has been reported most frequently in patients with sickle cell disease, however the prevalence of PAH is not yet clearly established. The prevalence of PH in sickle cell disease is undoubtedly much lower than 32% as suggested by echocardiography [47]. Recently, a prospective epidemiologic studies using echocardiographic screening and direct hemodynamic confirmation were conducted in 398 outpatients with sickle cell disease at referral centers in France [52]. In this study, the prevalence of a tricuspid regurgitant jet velocity of at least 2.5 m per second measured by echocardiography was 27%. In contrast, the prevalence of pulmonary hypertension confirmed on catheterization was only 6%, suggesting that echocardiographic evaluation alone had a low positive predictive value for PH in this population. Indeed, the precise mechanism of PAH in sickle cell disease remains uncertain.

### Group 1’: Pulmonary veno-occlusive disease and/or pulmonary capillary hemangiomatosis

PVOD and PCH are uncommon conditions, but they are increasingly recognized as causes of PH [53]. A recent clinicopathologic study [54] analyzed specimens from 35 patients diagnosed as having either PVOD (n = 30) or PCH (n = 5). PCH was identified in 24 (73%) cases diagnosed as PVOD. Indeed, venous involvement was present in 4/5 cases initially diagnosed as PCH. These findings suggest that PCH may be an angioproliferative process frequently associated with PVOD. Similarities in pathologic features and clinical presentation suggest that these disorders may be two different presentation of the same disease [54].

Although PVOD and PCH may present similarly to idiopathic PAH, there are a number of important differences. These include the presence of crackles on examination, radiologic abnormalities on high-resolution computed tomography of the chest (ground glass opacities, septal thickening, mediastinal adenopathy) [55-58], hemosiderin-laden macrophages on bronchoalveolar lavage [59], and a lower DLCO and PaO_2_ in patients with PVOD or PCH [58]. PVOD/PCH remains a difficult disorder to categorize, as it shares characteristics with idiopathic PAH but also has a number of distinct differences. Given the current evidence, it was decided that PVOD/PCH should be a distinct category but not completely separated from PAH and PVOD/PCH are designated as 1’ in the current classification.

### Group 2: Pulmonary hypertension due to left heart disease

Left-sided ventricular or valvular diseases may produce an increase of left atrial pressure, leading to a backward transmission of the pressure and a passive increase of pulmonary arterial pressure. Left heart disease, probably represents the most frequent cause of PH [60]. In this situation, PVR is normal or near normal (<3.0 Wood units) and there is no gradient between mean PAP and pulmonary wedge pressure (transpulmonary gradient <12 mm Hg). In the Dana Point classification, the increasing recognition of left-sided heart dysfunction with preserved ejection fraction leads to changes in the subcategories of Group 2 and now this group include three distinct etiologies: left heart systolic dysfunction, left heart diastolic dysfunction, and left heart valvular disease. In some patients with left heart disease, the elevation of pulmonary arterial pressure is out of proportion to that expected from the elevation of left arterial pressure (transpulmonary gradient >12 mm Hg), and PVR is increased to >3.0 Wood units (19–35% of patients) [60]. However, there is no widely accepted hemodynamic definition of transpulmonary gradient, and future recommendations may propose new definition and threshold of this gradient. Some patients with left heart valvular disease or even left heart dysfunction can develop severe PH of the same magnitude as that seen in PAH [61-63]. The elevation of PAP and PVR may be due to either the increase of pulmonary artery vasomotor tone and/or pulmonary vascular remodeling [64,65].

### Group 3: Pulmonary hypertension due to lung diseases and/or hypoxia

In this group, the predominant cause of PH is alveolar hypoxia as a result of either chronic lung disease, impaired control of breathing, or residence at high altitude. However, the precise prevalence of PH in all these conditions remains largely unknown. In the revised classification, the heading has been modified to reinforce the link with the development of PH. A category of lung disease characterized by a mixed obstructive and restrictive pattern was added, including chronic bronchiectasis, cystic fibrosis and the recently described syndrome of combined pulmonary fibrosis and emphysema in which the prevalence of PH is almost 50% [66,67]. In PAH associated with parenchymal lung disease, the increase of pulmonary arterial pressure is usually modest (mean PAP lower than 35 mmHg) [68]. Interestingly, in some patients, increase of PAP is out of proportion and be higher than 35 mmHg [69]. In a retrospective study of 998 patients with chronic obstructive pulmonary disease who underwent RHC, only 1% had severe PH [70]. These patients with more severe PH were characterized by mild-to-moderate airway obstruction, severe hypoxemia, hypocapnia, and a very low diffusing capacity for carbon monoxide.

### Group 4: Chronic thromboembolic pulmonary hypertension

Chronic thromboembolic pulmonary hypertension (CTEPH) was included in Group 4. The incidence of CTEPH is uncertain. CTEPH represents however a frequent cause of PH and occurs in up to 4% of patients after an acute pulmonary embolism [71,72]. In the classification from the third World Symposium on PH, CTEPH was divided into 2 subgroups: proximal CTEPH and distal CTEPH, depending on the feasibility of performing pulmonary thromboendarterectomy. Currently, there is no consensus about the definitions of proximal and distal CTEPH and the decision of surgery may vary depending on individual centers [73]. Thus, the Dana Point Classification propose only one group irrespectively of proximal or distal obstruction. Patients with suspected or confirmed CTEPH need to be referred to expert centers with experience in the management of CTEPH, to consider the feasibility of performing surgery. The indication for surgery depends on the location of the obstruction, the correlation between hemodynamics and the degree of obstruction assessed by angiography, comorbidities, the willingness of the patient, and the experience of the surgeon [74,75]. Patients who are not candidates for surgery may benefit from PAH-specific medical therapy [76,77]; however, further evaluation of these therapies in randomized control trials are needed [78].

### Group 5: PH with unclear or multifactorial etiologies

#### *Group 5.1 Hematologic disorders*

PH has been reported in chronic myeloproliferative disorders including polycythemia vera, essential thrombocythemia, and chronic myeloid leukemia [79,80]. Several mechanisms may be implicated in PH associated with chronic myeloproliferative disorders including high cardiac output, asplenia, direct obstruction of pulmonary arteries by circulating megakaryocytes [81], CTEPH [82], portal PH, and congestive heart failure. Splenectomy as a result of trauma or as a treatment for hematologic disorders may increase the risk of developing PH [83]. As described above, dasatinib use may be one cause of PAH, particularly in chronic myeloid leukemia [23].

#### *Group 5.2 Systemic disorders*

The second subgroup includes systemic disorders, including sarcoidosis, pulmonary Langerhans cell histiocytosis, lymphangioleiomyomatosis, neurofibromatosis or vasculitis.

PH is a well recognized complication of sarcoidosis [84], with a reported prevalence of 1–28% [84]. PH is multifactorial and usually attributed to the destruction of capillary bed by the fibrotic process and/or the result of chronic hypoxemia [85]. However, the severity of PH is frequently out of proportion to the degree of parenchymal lung disease and blood gas abnormalities, suggesting specific pulmonary vascular involvement [86]. Such mechanisms may include extrinsic compression of large pulmonary arteries by lymph node enlargement, and granulomatous infiltration of the pulmonary vasculature, especially the pulmonary veins, which sometimes mimic PVOD [87].

Pulmonary Langerhans cell histiocytosis is an uncommon cause of infiltrative and destructive lung disease. Severe PH is a common feature in patients with end stage disease [88] and PH in these patients is usually related to chronic hypoxemia and/or abnormal pulmonary mechanics. Histopathologic examination has shown severe diffuse pulmonary vasculopathy involving predominantly intralobular pulmonary veins and, to a lesser extent, muscular pulmonary arteries [89].

Lymphangioleiomyomatosis is a rare multisystem disorder predominantly affecting women, characterized by cystic lung destruction, lymphatic abnormalities, and abdominal tumors. PH is relatively uncommon in patients with lymphangioleiomyomatosis [90]. Recently, a series of 20 cases of lymphangioleiomyomatosis associated PH has been reported showing that PH is usually moderate in this setting and associated with pulmonary function impairment [91].

Neurofibromatosis type 1, also known as von Recklinghausen’s disease, is an autosomal-dominant disease. The disease is occasionally complicated by systemic vasculopathy. A series of cases of PH have been reported in the setting of Neurofibromatosis type-1 [92].

#### *Group 5.3 Metabolic disorders*

PH has been reported in a few cases of type Ia glycogen storage disease, a rare autosomal-recessive disorder caused by a deficiency of glucose-6-phosphatase [93-95]. The mechanisms of PH are uncertain but portocaval shunts, atrial septal defects, severe restrictive pulmonary function defects or thrombosis are thought to play a role. In one case, autopsy findings revealed the presence of plexiform lesions [96].

Gaucher’s disease is a rare disorder characterized by a deficiency of lysosomal B glucosidase, which results in an accumulation of glucocerebroside in reticuloendothelial cells. In a study of 134 patients with Gaucher’s disease who were systematically screened by echocardiography, PH was not uncommon [97]. In this setting, several potential mechanisms for PH have been suggested, including interstitial lung disease, chronic hypoxemia, capillary plugging by Gaucher cells and splenectomy [97,98].

The association between thyroid diseases and PH has been reported in some studies [99]. A prospective study of 63 consecutive adult patients with PAH found a prevalence of autoimmune thyroid disease, including both hypothyroidism and hyperthyroidism, in 49%, suggesting that these conditions may share a common immunogenetic susceptibility [100].

#### *Group 5.4 Miscellaneous conditions*

The last subgroup includes a number of miscellaneous conditions, including tumoral obstruction, fibrosing mediastinitis or chronic renal failure on dialysis.

A progressive obstruction of proximal pulmonary arteries leading to PH may be observed in tumor obstruction when a tumor grows into the central pulmonary arteries with additional thrombosis. Such cases are due principally to pulmonary artery sarcomas, which occur rarely but are usually rapidly fatal [101-103]. The differential diagnosis with CTEPH can be difficult and CT angiography may be useful to differenciate an obstruction by tumor or thrombotic material. Occlusion of the microvasculature by metastatic tumor emboli represents a cause of rapidly progressive PH.

Fibrosing mediastinitis have been mainly reported in sarcoidosis, tuberculosis, histoplasmosis and after radiotherapy. Fibrosing mediastinitis may be associated with severe PH due to compression of both pulmonary arteries and veins.

Lastly, PH has been reported in patients with end-stage renal disease maintained on long-term hemodialysis. Based on echocardiographic studies, the prevalence of PH in this patient population is estimated up to 40% [104]. PH in these patients may be explained by high cardiac output (resulting from the arteriovenous access, anemia and fluid overload) and potential diastolic and systolic left heart dysfunctions. Furthermore, hormonal and metabolic modification associated with end-stage renal disease might lead to dysfunction of normal pulmonary vascular tone.

## Epidemiology

Information relative to the natural history of PAH was derived from a national registry conducted in the USA in the early 1980s, which included 187 patients with idiopathic PAH followed for up to 5 years. This study characterized the disease and confirmed its poor prognosis with a median survival of 2.8 years [105,106]. Better understanding of pathophysiological mechanisms of PAH has led to the development of novel therapeutic strategies in the last decade which improved quality of life, exercise capacity and survival of these PH patients [107].

In France, a national multicenter registry prospectively collected data from 674 adults with PAH from October 2002 to October 2003 and followed these patients during a 3-year period [108]. This prospective study has shown more up to date data on the epidemiology of PAH. This registry has confirmed the female predominance in most subtypes of PAH with a female/male sex ratio (SR) of 1.9 for all PAH patients, and particularly in idiopathic PAH (SR 1.6), familial PAH (SR 2.2) and anorexigen-associated PAH (SR 14.9) [108]. The mean age of PAH patients in this cohort was 50 years with a quarter of patients older than 60 years underlining the possibility of developing PAH in all ages. In this assessed cohort, 39.2% of patients had idiopathic PAH and 3.9% familial PAH. In the subgroup of PAH, where PAH was associated with other conditions, 15.3% had connective tissue disease, 11.3% congenital heart diseases, 10.4% portal hypertension, 9.5% anorexigen-related PAH and 6.2% HIV infection [108]. In France, the low estimation of the prevalence of PAH and idiopathic PAH are respectively of 15 cases and 5.9 cases/million adult inhabitants. The low estimation of PAH incidence is 2.4 cases/million adult inhabitants/year. This study conducted five years after the withdrawal of fenfluramine derivatives underlines that anorexigen-associated PAH remains an important medical problem nowadays.

Recent prospective data in consecutive patients with idiopathic, familial or anorexigen-associated PAH patients followed-up for a period of three years (56 incident and 298 prevalent cases) showed better survival rates than historical cohort. For incident cases, estimated survival rates were 85.7%, 69.6% and 54.5% at one, two and three year of follow-up, respectively. In combined mixed population (incident patients and prevalent patients diagnosed within three years before study entry), estimated one, two and three year survival was 82.9%, 67.1% and 58.2%, respectively. Parameters significantly associated to improved survival were female gender, preserved 6-MWD and normal cardiac output on RHC [109].

Data from the REVEAL registry (US Registry to Evaluate Early and Long-Term PAH Disease Management) are similar concerning the one year survival rate estimated to 91%. Variables independently associated with increased mortality included pulmonary vascular resistance >32 Wood units, NYHA functional class IV, men older than 60 years and familiy history of PAH [110].

## Genetics

It is now well known that PAH can be either sporadic or clustered in families [111-114]. In 1954, Dresdale published a detailed description of a family that included three related subjects with severe PAH of unknown aetiology. Because of physician’s awareness of the familial occurrence of the disease, other cases of familial PAH were described. The National Institute of Health Registry (NIH Registry) provided the first estimate of this condition, and evaluated that at least 6% of individuals diagnosed with sporadic PAH have a family history of the disorder. In the French PAH registry, 26 cases of familial PAH were identified in the cohort of 674 patients, which corresponds to a prevalence of 3.9% of all cases of PAH and a proportion of 7.3% of familial PAH in the subgroup of idiopathic, familial or anorexigen-associated PAH [108]. Studies of genealogies of familial PAH advanced our understanding that the disease segregates an autosomal dominant trait with a markedly reduced penetrance, since only 10-20% of necessary carriers of the mutation will develop PAH [115,116]. In 2000, linkage analysis in PAH affected families found mutations within the *bone morphogenetic protein receptor type 2 gene (BMPR2) *[117,118]. *BMPR2* gene encodes for a type 2 receptor member of the transforming growth factor beta (TGF-β) family. Nowadays, germline *BMPR2* mutations are detected in 58-74% of PAH patients with a family history of the disease, and in 3.5-40% of so called idiopathic PAH patients [6,11,108,119-123]. The observation of the development of PAH in patients displaying hereditary hemorrhagic telangiectasia (HHT), an autosomal dominant vascular dysplasia, allowed us to identify two other PAH predisposing genes: *ACVRL1 (Activin A receptor type II-like kinase 1*) and *ENG* (*endoglin*) genes. Mutations in these two genes are infrequent in PAH but are frequently identified in HHT [120,124-130]. Moreover, recent reports described two PAH patients carriers of a mutation in Smad8 gene, one PAH patient carrier of a Smad1 mutation and one PAH patient carrier of a Smad5 mutation [8,9]. *BMPR2 gene, ACVRL1, ENG*, Smad8, Smad1 and Smad5 genes are encoding proteins which are involved in the TGF-β signaling pathway. More recently, using whole exome sequencing in a large PAH family, Austin et al. demonstrated the involved of mutations in *caveolin-1* gene in the development of PAH [10] They confirmed their findinds identifying a *caveolin-1* mutation in an unrelated PAH patient. Caveolin-1 protein is necessary for the formation of calveola, which are crucial for joining membrane receptors and initiating cellular signaling cascade such as TGF-β signalling pathway. This supports the hypothesis that mutations in genes involved in the TGF-β signaling pathway may be a trigger for pulmonary vascular remodeling. Moreover, this signaling pathway controls growth, differentiation and apoptosis of various cell types like pulmonary vascular endothelial cells (ECs) and smooth muscle cells (SMCs). Thereby, mutations in genes involved in the TGF-β signaling pathway may be responsible for abnormal proliferation of pulmonary vascular SMCs and may promote ECs apoptosis, which might lead to the selection of apoptosis resistant cells and formation of plexiform lesions, the hallmark of idiopathic PAH [131,132].

The analysis of clinical, functional and hemodynamic characteristics of PAH patients revealed that patients carriers of a *BMPR2* or an *ACVRL1* mutation are younger at diagnosis than patients with idiopathic PAH. In addition, they have more severe hemodynamic parameters at diagnosis [114,120]. 

Several lines of evidence point to the potential requirement of additional factors, either environmental or genetic, in the pathogenesis of the disease. As mentioned, Humbert and co-workers have shown that exposure to fenfluramine derivatives greatly increase the risk of developing severe PAH in patients with *BMPR2* mutations [111]. Moreover, modified genes could participate or facilitate the development of PAH. In fact, recent studies have suggested the potential role of serotonin transporters, serotonin receptors, potassium channels, or angiopoietin-1 [133]. Finally, PAH mostly occurs in females irrespective of *BMPR2* mutation status [134]. To explain overrepresentation of PAH female patients, it was suggested that estrogen metabolism might participate in the pathogenesis of PAH [135,136].

## Pathophysiology

**Figure 1:**
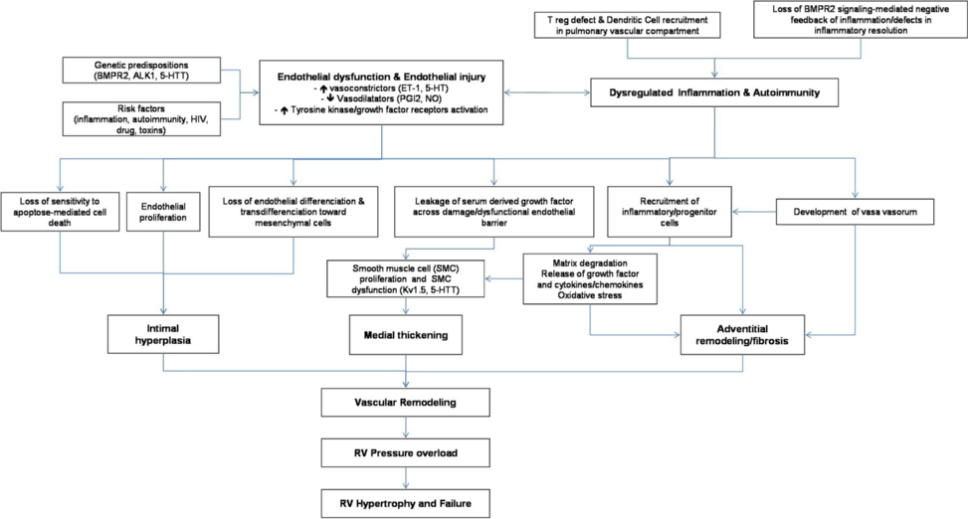
**Pathophysiology of PAH.** The pulmonary vascular remodeling responsible for PAH is the consequence of closely intertwined predisposing and acquired factors. Thoses pathological elements affect all three layers of precapillary pulmonary arteries leading to intimal hyperplasia, medial thickening and adventitial remodeling/fibrosis. Intra- but also extra-pulmonary cells, such as inflammatory and progenitor cells, are suspected to play a role in this remodeling. This increases right ventricular afterload and consequently results in right ventricular failure.

PAH is a disease which affects small pulmonary arteries. It is characterized by vascular obstruction leading to progressive increase in vascular resistance. This increases right ventricular afterload and consequently results in right ventricular failure. Intima and media proliferation and its consequent pulmonary vascular obstruction are considered to be the key element in the pathogenesis of PAH. Vasoconstriction, vascular remodeling and thrombosis are factors that increase pulmonary vascular resistance in PAH [107,137]. These processes involve a multitude of cellular and molecular elements (Figure 1).

### Cellular factors

Proliferation of smooth muscular cells in the small peripheral pulmonary arteries is a common characteristic in all forms of PAH. In hypoxic models, fibroblasts of the adventitia migrate to the media and intima, where proliferation and production of matrix proteins are observed [138]. Neovascularization, mainly of the adventitia, occurs concomitantly to the thickening of the vascular walls [139].

In response to certain stimuli, endothelial cells abnormally proliferate to form plexiform lesions in several forms of PAH. Plexiform lesions consist of endothelial cells, matrix proteins and fibroblasts and obliterate the vascular lumen [140]. The stimuli for endothelial proliferation is still unknown but several factors have been incriminated such as hypoxia, inflammation, shear stress, drugs, viral infections and genetic susceptibility. Extrapulmonary cells may also participate in the vascular remodeling responsible for PAH. Indeed, fibrocytes and c-kit + cells are mobilized from the bone marrow, and may differentiate into vascular cells and/or produce pro-angiogenic factors to participate in the pathogenesis of PAH [141,142]. The CXCL12/CXCR4 axis may play an important role in the pulmonary recruitment of these circulating progenitors and can be therapeutically targeted [143].

Inflammatory mechanisms seems to play an important role in certain forms of PAH such as PAH associated with auto immune diseases or HIV infection [144]. In fact, in severe cases of PAH associated with systemic lupus erythematosus disease, some patients improved both clinically and hemodynamically with administrated immunosuppressant treatment. Thirty to 40% of patients with PAH have circulating auto-antibodies and elevated plasma concentrations of pro-inflammatory cytokines such as interleukin 1 (IL-1) and interleukin-6 (IL-6), and chemokines such as fractalkine and MCP-1 [145,146]. Inflammatory cells, such as lymphocytes B and T, macrophages, mastocytes and dendritic cells, can also be found in plexiform lesions of severe PAH [147,148]. Chemokines, like RANTES and fractalkine are also overly expressed in the pulmonary vascular endothelium of PAH patients [145].

Thrombosis and platelet dysfunction can be important in the development of PAH. Abnormalities of thrombosis, endothelial cells or platelets can generate or aggravate thrombosis in situ. Elevated plasma concentrations of D-dimers and fibrinopeptides A and B, in certain patients with PAH, are the proof of an abnormal intravascular coagulation process. Elevated plasma concentrations of von-Willebrand factor and plasminogen activator inhibitor type 1 also reflect endothelial dysfunction in PAH. It has been demonstrated that shear stress creates pro-thrombotic vascular lesions in PAH that may lead to thrombosis in situ. But platelet function is not limited to coagulation. In response to certain stimuli, platelets can produce prothrombotic, vasoactive or mitogenic factors, such as thromboxane A2 (TXA2), platelet-derived growth factor (PDGF), serotonin (5-hydroxytryptamine, 5-HT), transforming growth factor beta (TGF-β) and vascular endothelial growth factor (VEGF) that participate in vasoconstriction and vascular remodeling [149,150].

### Autoimmunity and PAH

The self-tolerance is controlled in the periphery by a particular population of T-lymphocytes called regulatory T-lymphocytes (Treg). The breakdown of self-tolerance can lead to the development of an autoimmune response (i.e. directed against self antigens) that can finally give rise to an autoimmune disease. Huertas et al. [151] showed that circulating Treg number was comparable in idiopathic PAH and SSc-PAH patients. However the percentage of those expressing leptin receptors was higher in idiopathic PAH and SSc-PAH as compared to controls, and their function was reduced in idiopathic PAH and SSc-PAH patients as compared to controls in a leptin-dependent manner [151]. Work on chronic inflammatory disorders and autoimmune diseases suggest that pathogenic antibodies and T cells may be generated locally, in the targeted organ, in highly organized ectopic lymphoid follicles commonly called tertiary lymphoid tissues. Recently, Perros et al. [152] described the presence of highly organized perivascular follicles in idiopathic PAH lungs arguing for specific immune-adaptive mechanisms in the pathophysiology of the disease. One can propose that deregulated and unresolved pulmonary inflammation on the background of a genetic predisposition, could result in persisting vascular remodelling leading to PAH. An initial acute inflammation that is normally expected to resolve with return to homeostasis, could conduct the production of auto-antibodies against vascular wall components, and would shift to chronic persisting and chronic inflammation, endothelial barrier breakdown, infiltration by immune cells, local and chronic autoimmunity, and vascular remodeling culminating in PAH.

### Molecular factors

Many authors consider pulmonary vasoconstriction as an early event in the process of PAH. Vasoconstriction has been associated with an abnormal function or expression of potassium channels and with endothelial dysfunction [107]. Endothelial dysfunction results in a decreased production of vasodilators such as nitric oxide (NO) and prostacyclin and an increased production of vasoconstrictors such as endothelin-1 [153].

Prostacyclin (prostaglandin I2) is a potent pulmonary vasodilator that acts via the cyclic adenosine monophosphate (cAMP) pathway. It inhibits the proliferation of smooth muscle cells and decreases platelet aggregation. Production of prostacyclin is reduced in endothelial cells of patients with PAH [154]. PAH therapy based on prostacyclin and its derivates have proven efficacy both hemodynamically and in clinical trials. NO is also a pulmonary vasodilator which acts via the cyclic guanosine monophosphate (cGMP) pathway. To increase pulmonary vasodilatation dependant on NO, a recent therapeutic strategy has targeted type 5 phosphodiesterase which degrades cGMP. Sildenafil or tadalafil, type 5 phosphodiesterase inhibitors, have proven their efficacy in patients with PAH [155]. Vasoactive intestinal peptide (VIP) is a neurotransmitter that has systemic and pulmonary vasodilator properties. It also inhibits smooth cell proliferation and decreases platelet aggregation and acts via the activation of the cAMP and cGMP systems [156]. Low plasmatic concentrations of VIP have been measured in pulmonary arteries of patients with PAH.

Endothelin-1 (ET-1) is an endothelially-derived peptide that has two receptor subtypes, designated as endothelin A (ETRA) and endothelin B (ETB), located on smooth muscle cells of pulmonary arteries. By ligating the ETRA, ET-1 intracellular calcium concentrations increase and activates the protein kinase C pathway [157]. ET-1 is a potent pulmonary vasoconstrictor and stimulates mitosis of arterial smooth muscle cells, thus contributing to pulmonary vascular remodeling. Pulmonary and plasma levels of ET-1 are elevated in human PAH and in experimental animal models of PAH [158]. The therapeutic efficacy of endothelin receptor antagonists (Ambrisentan, Bosentan) has been demonstrated in clinical trials in the pathophysiology of PAH.

In hypoxic models of PAH, hypoxia inhibits one or several voltage dependant potassium channels of the pulmonary arterial smooth muscle cells. This leads to membrane depolarization and opening of voltage dependant calcium channels with a subsequent increase of the intracellular calcium concentration and cellular contraction. Certain potassium channels are under expressed in pulmonary artery smooth muscle cells of patients with PAH [159,160]. It is still unknown whether abnormalities of the potassium channels are acquired or genetic. However, it has been demonstrated that anorexigens, such as dexfenfluramine and aminorex, directly inhibit certain potassium channel subtypes [161]. Certain medications such as dichloroacetate and sildenafil increase the expression and function of potassium channels.

In PAH, plasmatic concentrations of serotonin (5-hydroxytryptamine, 5-HT) are elevated [150]. An association between anorexigens and serotonin was established in the 1960s. Aminorex and fenfluramin both increase plasmatic levels of serotonin. The variability of the expression and activity of the transporter of 5-HT (5-HTT) contributes to pulmonary vascular remodeling in human and experimental models of PAH [162]. Some studies have shown that the serotonin selective reuptake inhibitor fluoxetin prevents the development of PAH in mice [163]. Some 5-HT receptor subtypes may also be implicated in the development of hypoxia induced PAH [164].

Rho proteins regulate fundamental cellular functions such as contraction, migration, proliferation and apoptosis. Several studies have implicated Rho protein A and Rho kinases in the vasoconstriction and vascular remodeling of PAH [165,166]. RhoA and Rho kinase activities are increased in idiopathic PAH, in association with enhanced RhoA serotonylation. Direct involvement of the 5-HTT/RhoA/Rho kinase signaling pathway in 5-HTT-mediated pulmonary artery-smooth muscle cell (PA-SMC) proliferation and platelet activation during PH progression identify RhoA/Rho kinase signaling as a promising target for new treatments against PH [167].

Hypoxia inducible factor-1 (HIF-1) is a transcription factor that principally regulates cellular adaptation to hypoxia but also regulates several genes implicated in angiogenesis, erythropoiesis, cellular metabolism and survival [168]. In experimental mice heterozygote for the gene coding for HIF-1 alpha, hypoxia induced right ventricular hypertrophy, right ventricular pressure and medial thickening of pulmonary arterioles are reduced [169]. In immunohistological analysis of human plexiform lesions of patients with severe PAH, there was an overexpression of HIF-1 alpha in proliferating endothelial cells [170].

In conclusion, the pathophysiology of PAH is heterogeneous and multifactorial. The genetic mutations found in familial PAH and in a proportion of sporadic PAH are neither necessary nor sufficient for the development of PAH. Therefore, the current hypothesis is that of a genetic predisposition for PAH followed by a superimposed environmental factor (infection, inflammation, autoimmunity). Our understanding of the underlying pathophysiological mechanisms of PAH has lead to the development of new treatments such as prostacyclin analogues, endothelin receptor antagonists and type 5 phosphodiesterase inhibitors. However, future progress is still necessary in order to discover new pathophysiological pathways and to develop new therapeutic strategies in PAH.

## Histopathology: vascular changes

Vascular remodelling in pulmonary arterial and venous hypertension typically involves the small pulmonary vessels. Muscular arteries of less than 500 μm display a thickening within the intimal, medial and adventitial compartment. When involved, pulmonary veins of the interlobular septa and smallest preseptal venules show fibrous obliteration and / or muscularization. Even the smallest vascular level may be involved: patients with pulmonary venous hypertension frequently present focal proliferation of alveolar capillaries. The different histological pattern is presented here below.

### Arterial lesions

#### *Isolated medial hypertrophy*

**Figure 2:**
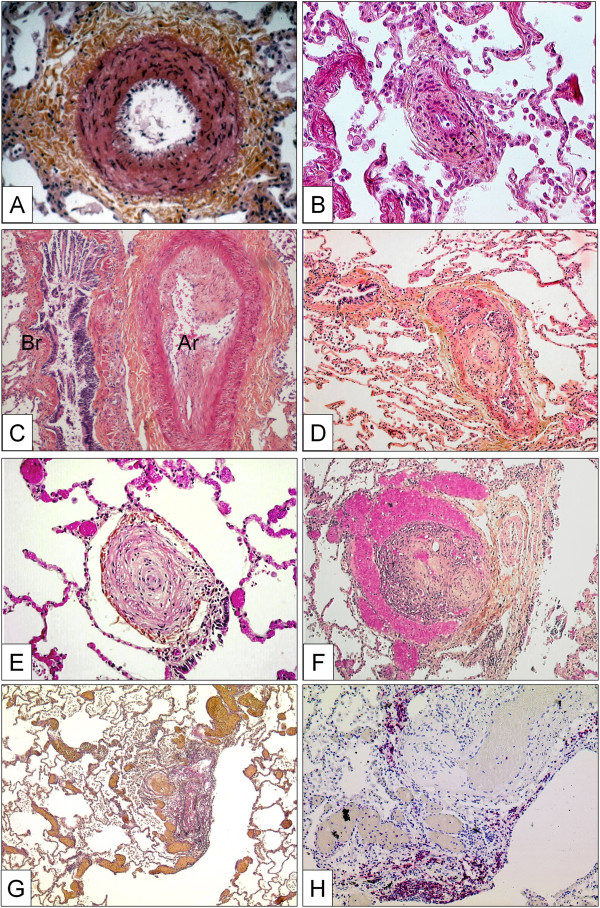
**Pulmonary arteries of the muscular type displaying obstructive arteriopathy in lungs of patients with PAH. A** Medial hypertrophy with smooth muscle cell proliferation and pronounced adventitial fibrosis. Magnification x200, Weigert-hematoxylin-phloxine-saffron staining (WHPS). **B** Concentric non-laminar intimal fibrosis comprising numerous myofibroblasts (arrows). **C** Eccentric intimal fibrosis corresponding to organized thrombotic material. Br: bronchus, Ar: pulmonary artery. Magnification x100, HES staining. **D** Thrombotic lesion, so called "colander-like lesion", with partial recanalization by microvessels. Note the similarity to plexiform lesions (F). Magnification x100, HES. **E** Concentric laminar intimal fibrosis, so called „onion-skin lesion“. Magnification × 200, HES. **F** Plexiform lesion with proliferation of small sinusoid-like vessels on a fibrotic matrix. Note surrounding dilated vessels. Magnification x100, HES. **G** Multiple dilation lesions being the sentinel of the centrally located plexiform lesion. Magnification × 40, Elastica-van-Gieson staining (EvG). **H** The same plexiform lesion after immunohistochemical staining with anti-CD3, a T-lymphocytic marker. Note the perivascular distribution of the inflammatory infiltrate. Magnification x100.

This abnormality of the vessel wall can be observed in all subgroups of PAH and may even be encountered in other forms of PH, e.g. in mitral valve stenosis. The lesion corresponds to a smooth muscle cell proliferation and / or recruitment within the tunica media; the histological criterion of hypertrophy / hyperplasia is fulfilled, when the diameter of a single medial layer, delimited by its internal and external elastic lamina, exceeds 10 per cent of the arteries cross-sectional diameter (Figure 2A). Isolated hypertrophy of the medial layer may be considered as an early and even reversible event as it has been shown in PH due to hypoxia in high altitude [171]. However, medial hypertrophy is usually associated with other PAH-lesions.

#### *Concentric and eccentric non-laminar intimal fibrosis*

Fibrotic lesions of the intimal layer are frequent in PAH-diseased lungs. The intima may be thickened by proliferation and recruitment of fibroblasts, myofibroblasts and other connective tissue cells, and consequently by the interstitial deposition of collagen (Figure 2B, C). In a purely descriptive approach, this thickening may be uniform and concentric, or focally predominating and eccentric. However, the eccentric intimal thickening is frequently observed in cases with thrombotic events and could represent residues of wall-adherent, organized thrombi. Thrombotic lesions, or so called in situ thrombosis, are a frequent pattern in different PAH-subgroups: organization and recanalization of totally occluding thrombotic material may lead to bizarre, fibrotic multi-channel lesions (so called “colander-like” lesions) which can be easily confounded with proliferative complex lesions (see below) (Figure 2D). Frequently, adventitial fibrosis is associated to intimal modifications (Figure 2A, B).

#### *Concentric laminar intimal fibrosis*

This morphologically conspicuous phenotype of intimal fibrosis is also known as “onion-skin” or “onion-bulb” lesion. Numerous concentrically arranged fibrotic layers occlude the arterial lumen of small (diameter: 100–200 μm) arteries (Figure 2E). The scary, cell-lacking morphology of this lesion is frequently found in patients suffering from idiopathic PAH and PAH associated to CTD [172]. Nevertheless, immunohistochemical analysis reveals fibroblasts, myofibroblasts and smooth muscle cells.

#### *Complex lesions*

The pathological classification of the World Symposium meetings in Venice and in Dana Point comprises three patterns, plexiform lesion, dilation lesion and arteritis. The plexiform lesion probably represents the most illustrious form of vascular lesions in PAH and affects various vascular compartments: focal intimal thickening of small pulmonary arteries, preferably beyond branching points and exuberant endothelial cell proliferation, leading to the formation of capillary-like, sinusoidal channels on a smooth muscle cell and collagen-rich matrix within the native arterial lumen and resulting in obstruction [173,174]. This glomeruloid-like arterial zone feeds into dilated, vein-like congestive vessels, which are perceivable at low magnification (Figure 2F). The latter vein-like vessels are also known as dilation lesions and may predominate the histological pattern (Figure 2G). Classical arteritis with transmural inflammation and fibrinoid necrosis, as first described by Heath and Edwards for PAH associated to congenital cardiac disease (Eisenmenger), is not a regular finding in PAH [175]. Nevertheless, perivascular inflammatory infiltrates of diseased pulmonary arteries in PAH-patients, consisting mainly of T- and B-lymphocytes, dendritic cells, mast-cells and macrophages can be regularly found [141,148,176] (Figure 2H). It has not been elucidated until now, whether this inflammatory pattern is of pathogenetic importance, or if it represents a pure epiphenomenon within disease evolution. The reported evidence of proinflammatory mediators, so called chemokines, released by altered endothelial cells of PAH-lungs strongly indicates a self-supporting and self-amplifying process [145,177].

#### *Venous and venular lesions (Pulmonary veno-occlusive disease and pulmonary hemangiomatosis)*

**Figure 3:**
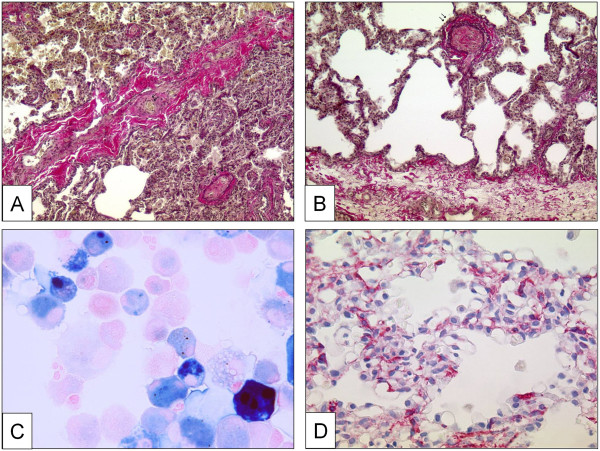
**Pulmonary veins with obstructive venopathy in lungs of patients with PVOD and a case of pulmonary capillary hemangiomatosis. A** Longitudinally dissected septal vein with asymmetric intimal and partially occlusive fibrosis. Note the intra-alveolar hemorrhage due to the post-capillary block on the upper half of the photograph. Magnification × 100, EvG. **B** Pre-septal venule with occlusive intimal fibrosis. Magnification × 100, EvG. **C** Bronchio-alveolar lavage in a PVOD-patient. Perls-Prussian-Blue staining. Note the siderophages displaying gradually different color-shades (see text). Magnification × 400. **D** Excessively proliferating alveolar capillaries in a patient with pulmonary capillary hemangiomatosis. Note protrusion of ectatic lumina into the alveoli. Magnification × 200, anti-CD31 staining.

A clear-cut differentiation between pre-and post-capillary pulmonary vascular lesions is sometimes difficult to make: lesions frequently concern veins and arteries in lungs of patients with PVOD, and vice-versa veins may be strongly involved in some subgroups of PAH. This is not contradictory because the clinical approach to ‘difficult-to-treat’ PAH-groups may be similar to clinical management of PVOD and PVOD-patients are frequently treated – under great precaution – with pulmonary arterial dilators, e.g. prostanoids. Recent reports, for example, indicate that CTD associated PAH, classically being considered as pre-capillary PH, simultaneously displays a PVOD-like pattern in histology [178,179]. Like in PVOD, the observed post-capillary lesions concern septal veins and pre-septal venules and usually consist of a loose and pauci-cellular and cushion-like intimal fibrosis that may totally occlude the lumen. A muscularization of both, septal veins and pre-septal venules may be observed (Figure 3A, B). Importantly, occult pulmonary hemorrhage regularly occurs in patients displaying PVOD. This particularity, which is certainly due to the post-capillary bloc, is of diagnostic importance, as bronchio-alveolar lavage can reveal an occult hemorrhage. The degree of hemorrhage can be evaluated semi-quantitatively and qualitatively using the Golde Score, which takes number and Perls-Prussian-Blue staining-degree of intra-alveolar siderophages into consideration (Figure 3C) [180]. Pulmonary capillary hemangiomatosis has been historically described as an aggressive capillary proliferation with patchy distribution within the pulmonary parenchyma: alveolar septa are thickened by 3 to 4 capillary layers, and infiltration of venous and bronchiolar structures with secondary occlusion may be present (Figure 3D). It is thought, that a clinically relevant post-capillary bloc is owed to this angiomatoid expansion. Occult hemorrhage or hemosiderosis, therefore, is frequently found [181]. However, Lantuejoul and co-workers have shown in a remarkable retrospective histological analysis, that capillary hemangiomatosis-pattern is virtually always present in PVOD, and vein-remodelling is constantly observed in case with a primary diagnosis of PCH [54]. The authors suggest the possibility, that PVOD and PCH might be the same disease, with a vein- or a capillary-predominating pattern. We fully support this view, and in our experience from the French National PH Reference Center, no clinical distinction is made between both conditions.

## Clinical description

### Symptoms and clinical signs of PH

There is no pathognomonic clinical sign of PH. Clinical presentation is related either to right heart failure or to associated diseases. Persistant dyspnea on exertion is the most frequent symptom; and it is present in almost patients even in the presence of mild hemodynamic abnormalities [1,182]. Dyspnea usually starts insiduously and is often neglected by patients which explain the delay of around two years in establishing the diagnosis of PH. The New York Heart Association (NYHA) provides a classification system for the clinicial evaluation of dyspnoea. Four categories are proposed to classify patients in functional classes (FC) based on how much they are limited during physical activity; the limitations/symptoms are in regard to normal breathing (Table 3).

**Table 3:** Modified New York Heart Association (NYHA) classification for pulmonary hypertension

CLASS I	Patients with pulmonary hypertension but without resulting limitation of physical activity. Ordinary physical activity does not cause undue dyspnoea or fatigue, chest pain, or near syncope.
CLASS II	Patients with pulmonary hypertension resulting in slight limitation of physical activity. They are comfortable at rest. Ordinary physical activity causes undue dyspnoea or fatigue, chest pain, or near syncope.
CLASS III	Patients with pulmonary hypertension resulting in marked limitation of physical activity. They are comfortable at rest. Less than ordinary activity causes undue dyspnoea or fatigue, chest pain, or near syncope.
CLASS IV	Patients with pulmonary hypertension with inability to carry out any physical activity without symptoms. These patients manifest signs of right heart failure. Dyspnoea and/or fatigue may even be present at rest. Discomfort is increased by any physical activity.

However, at time of diagnosis, 70% of patients are in NYHA FC III or IV. Chest pain, light-headedness and syncope may occur, particularly during physical efforts and are major signs of disease severity. Palpitations are frequent during physical efforts and may reveal true cardiac arrhythmias. Other symptoms of PAH include fatigue and weakness. Hemoptysis may complicate PAH and could be life-threatening, justifying embolization of dilated bronchial arteries. Hoarseness of the voice may occasionally be noted and is due to compression of the left laryngeal nerve by the dilated pulmonary artery (Ortner’s syndrome).

Signs of right heart failure may be observed in the most severe patients, including venous jugular distension, hepato-jugular reflux, hepatomegaly and hepatalgia. Lower limb edema, ascitis and generalized edema underscore the severity of right heart failure. Cardiac auscultation shows usually a prominent pulmonary component of S2, a systolic murmur of tricuspid regurgitation and more rarely a diastolic murmur of pulmonary regurgitation. Pulmonary auscultation is usually normal and contrasts with the importance of dyspnea. History and clinical examination should also screen for manifestations of extra thoracic diseases, particularly Raynaud’s syndrome which can be found in PAH associated with CTD and particularly in systemic sclerosis.

## Diagnostic methods

The diagnostic process of PAH requires a series of investigations that are intended to make the diagnosis, to clarify the clinical class of PH and the underlying type of PAH and to evaluate the functional and hemodynamic impairment [183]. The detection of PH requires investigations including electrocardiogram, chest radiograph and trans-thoracic echocardiogram. Other conditions which can induce PH will be identified by tests such as pulmonary function tests, arterial blood gases, ventilation and perfusion lung scan, high resolution computed tomography (HR-CT) of the chest and pulmonary angiography. Additional investigations are required for evaluation of PAH severity including exercise testing and hemodynamics. Additional imaging may clarify underlying lung abnormalities. Finally, right heart catheterisation confirms the definite diagnosis.

### Electrocardiogram (ECG)

The ECG may provide suggestive or supportive evidence of PH by demonstrating right ventricular hypertrophy and strain, and right atrial dilation. Right ventricular hypertrophy and right axis deviation are present in respectively 87% and 79% of patients with idiopathic PAH [105]. Unfortunately, the ECG has low sensitivity and specificity as a screening tool for detecting PH.

### Chest radiography

In 90% of idiopathic PAH patients, chest radiography is abnormal at the time of diagnosis [105]. Findings include central pulmonary arterial dilatation which contrasts with loss of the peripheral blood vessels. Right atrial and ventricular enlargement may be seen in more advanced cases. Chest radiography may help to identify associated moderate-to-severe lung disease or pulmonary venous hypertension due to left heart abnormalities.

### Pulmonary function test and arterial blood gases

Pulmonary function tests (PFT) will help to assess underlying lung abnormalities. Forced expiratory volume in one second (FEV1) and total lung capacity (TLC) in idiopathic PAH are usually normal or slightly abnormal. Low diffusing capacity of the lung for carbon monoxide (DLCO) has been reported in PAH patients, but is more pronounced in PVOD patients with often severe reductions under 50% of the predicted value [56,184]. Results of arterial blood gases usually show mild hypoxemia and hypocapnia. Severe hypoxemia may be a parameter of underlying PVOD or chronic lung disease.

### Exercise testing

The normal physiologic response of the pulmonary vasculature to exercise consists of distension of pulmonary arteries and arterioles as well as recruitment of previously unused vascular bed. Thus, in health, pulmonary artery pressure rises minimally in response to increased blood flow and pulmonary vascular resistance decreases because of the remodeled vasculature. These mechanisms are impaired in the course of PH. Cardiopulmonary exercise testing (CPET) has been shown to be useful in assessing the severity and prognosis of PAH [15,185]. Several mechanisms are associated: (1) failure to perfuse the ventilated lung, leading to an increase of physiologic dead space and ventilatory requirement; (2) failure to increase cardiac output appropriately in response to exercise, causing an early lactic acidosis, thereby increasing acid ventilatory drive; and (3) exercise-induced hypoxemia increasing the hypoxic ventilatory drive. The ventilatory expired gas abnormalities precipitated by PH are multifactorial and associated with disease severity. CPET assesses and measure the ventilation–perfusion mismatch (i.e. acceptable ventilation/diminished perfusion), reflected by an elevated VD/VT or VE/VCO2 ratio or slope and diminished partial pressure of end-tidal carbon dioxide (PETCO2), and the decreased peak VO2 and VO2 at the ventilatory threshold (VT). Peak VO2, VE/VCO2 ratio or slope or PETCO2, measure during CPET, all demonstrated independent and strong prognostic value as univariate markers [186].

### Transthoracic doppler-echocardiography (TTE)

TTE is a non-invasive screening test for patients with suspected PH. TTE estimates pulmonary artery systolic pressure (sPAP) and may provide additional information about the cause and consequences of PH. The estimation of PAP is based on the peak velocity of the jet of tricuspid regurgitation. The simplified Bernoulli equation describes the relationship of tricuspid regurgitation velocity and the peak pressure gradient of tricuspid regurgitation = 4 x (tricuspid regurgitation velocity). Estimation of PA systolic pressure require to take into account right atrial pressure (PA systolic pressure = tricuspid regurgitation pressure gradient + estimated right atrial pressure). Right atrial pressure cannot be measured and is estimated based on the diameter and respiratory variation of the inferior vena cava [15]. An alternative approach to echocardiographic diagnosis of PH is based on the comparison of tricuspid regurgitation velocity with values reported in a healthy population. Ideally, the influence of age, sex and body mass should be taken into consideration [187]. This method avoids cumulative error but is less directly linked to the accepted hemodynamic definition of PH based on mPAP [15].

Other echocardiographic variables that might raise or reinforce suspicion of PH independently of tricuspid regurgitation velocity should always be considered. They include an increased velocity of pulmonary valve regurgitation and a short acceleration time of RV ejection into the PA. Increased dimensions of right heart chambers, abnormal shape and function of the interventricular septum, increased RV wall thickness, pericardial effusion and dilated main PA are also suggestive of PH, but these signs are considered to be related to the hemodynamic severity [15].

Besides identification of PH, TTE also allows a differential diagnosis of the possible causes of pulmonary hypertension. TTE can recognize left heart valvular diseases and myocardial diseases responsible for post-capillary PH, and congenital heart diseases with systemic-to-pulmonary shunts. The venous injection of agitated saline can help to identify patent foramen ovale or small sinus venosus type atrial septal defects. Transesophageal echocardiography is rarely required in the setting of PH.

### Ventilation/perfusion lung scan

Ventilation/perfusion lung scan should be systematically assessed to screen for CTEPH. Indeed, V/Q lung scan is the method of choice to detect CTEPH [188] and normal V/Q scan can eliminate CTEPH.

### High resolution computed tomography of the chest

High resolution computed tomography of the chest (HRCT) supplies detailed information about underlying lung parenchyma disease, such as pulmonary emphysema or interstitial lung disease. A number of various pathologic features may be detected on chest HRCT including pericardial effusions and pulmonary artery enlargement, defined by the ratio of the diameter of main pulmonary artery to that of the thoracic aorta >1. In the setting of CTEPH, contrast HRCT of the pulmonary arteries may show changes like complete vessel obstruction, vessel cut-offs, intimal irregularities, incorporated thrombus formations as well as bands and webs [189]. Furthermore, collaterals from bronchial arteries can be identified with this technique. Proximal pulmonary obstruction is displaying about significant and accessible organized fibrous tissue in segmental or subsegmental arteries. If no proximal obstruction or obliteration is noted; lesions are considered to be distal, non-accessible to surgery intervention. In some cases, pulmonary angiography is necessary to differentiate between proximal or distal obstructions. Chest HRCT may also suggest PVOD in the presence of adenopathy mediastinal, ground glass opacities and septal lines [53].

### Pulmonary angiography

In CTEPH, pulmonary angiography may be helpful to determinate surgically accessible form. Typical angiographic findings in CTEPH are complete obstruction, band and webs as well as intimal irregularities [74]. Pulmonary angiography may be also helpful in the setting of fibrosing mediastinitis.

### Cardiac magnetic resonance imaging

Cardiac magnetic resonance imaging (MRI) allows non invasive evaluation of right ventricular size, morphology and shape. It provides information on right ventricular function and allows non-invasive assessment of blood flow including cardiac output, stroke volume, distensibility of pulmonary artery and right ventricular mass [190,191]. Decreased stroke volume, an increased right ventricular right ventricular end-diastolic volume and a decreased measured at baseline are associated with poor prognosis of disease [192,193]. In addition, it has been demonstrated that deterioration of these parameters at one-year follow-up were also predictors of mortality [193]. Thus cardiac MRI could represent a non-invasive tool to evaluate severity of PAH patients at baseline and during follow-up. Further studies are needed to evaluate the precise place of cardiac MRI in the management of PAH patients.

### Abdominal ultrasound scan

Abdominal ultrasound should be performed in all patients if PH is suspected to exclude portal hypertension or liver disease. When portal hypertension is suspected, the diagnosis can be confirmed during RHC by measurement of an increased gradient between the free and occluded hepatic vein pressure [194].

### Blood tests

Serological tests for HIV, hepatitis B or C serology should be performed to screen for associated diseases. The thyroid hormone measurement may reveal either hyperthyroid dysfunction or autoimmune thyroiditis, frequently encountered in PAH.

### Right heart catheterization

Invasive hemodynamic assessment with right heart catheterization is requested to confirm the diagnosis of PH showing a resting mPAP of ≥25 mmHg and a normal PCWP [2]. This value has been used for selecting patients in all RCTs and registries of PAH, however normal mPAP at rest is around of 14 mmHg, with an upper limit of normal of 20 mm Hg. The significance of a mean PAP between 21 and 24 mmHg is currently unclear. No definition of PH on exercise was currently adopted, because of the large variability of mPAP on exercise in healthy individuals.

The assessment of PCWP may allow the distinction between precapillary (normal PCWP ≤15 mmHg) and postcapillary PH (PCWP >15 mm Hg). In post-capillary PH, the last guidelines from the 4th World Symposium (Dana Point) proposed a dichotomy between "passive" and "reactive" (out-of-proportion) post-capillary PH based on transpulmonary pressure gradient (mPAP–PCWP, respectively ≤ or > 12 mm Hg). Indeed, there is no clear consensus on this definition and the future recommendations arising from the last World Symposium on PH (Nice, 2013) should propose new definition to define these two entities.

Measurement values obtained by RHC are PAP (diastolic, mean and systolic), right atrial pressure (RAP), PCWP, right ventricular pressure and cardiac output (CO) preferably by the thermodilution method. In contrast to the thermodilution method, the Fick method is mandatory in patients with suspected CHD. In experienced centres, RHC procedures have low morbidity and mortality rates [195]. Elevated mean right atrial pressure reduced CO and mixed venous oxygen saturation (SVO2) are related to the prognosis of PAH patients.

### Assessment of disease severity

NYHA functional class at baseline or after initiation of epoprostenol treatment, signs of right heart failure, 6-MWD, peak VO2, echocardiographic parameters, hemodynamic parameters and biological tests (hyperuricemia, brain natriuretic peptide, troponin) predict prognosis in idiopathic PAH when assessed at baseline.

Patients presenting PVOD or PAH associated with CTD (frequently associated with venous involvement) have a worse prognosis than patients with idiopathic PAH [53]. Patients with PAH associated with congenital systemic to pulmonary shunts have a more slowly progressive course than idiopathic PAH patients. Few data are available in other conditions such as HIV infection or portal hypertension. In these circumstances, underlying diseases may contribute to the overall outcome. In clinical practice, the prognostic value of a single variable in the individual patient may be less significant than the value of multiple concordant variables.

A score has been proposed (REVEAL Registry Risk Score) to evaluate severity of newly diagnosed PAH patients [196]. This score was based on several parameters including subgroups of PAH, renal insufficiency, age > 60 years, NYHA FC, systolic blood pressure, heart rate, 6-MWD, BNP, pericardial effusions, DLCO, RAP and PVR [196].

## Management of PAH

### General measures

#### *Physical activities*

Peripheral vasodilatation or increased cardiac demand will put PAH patient at risk of acute cardiac failure and syncope. Thus, we traditionally advise against extreme physical activity. Patients are taught to stay active while adapting effort according to their symptoms. Nevertheless, the appropriate level of physical activity is difficult to define. To date, only few studies have evaluated to effect of cardio-respiratory rehabilitation in PAH. One of this program involved three weeks of inpatient rehabilitation followed by three month training at home with phone call supervision. No modification on cardiac hemodynamic was observed on echocardiography but 6-MWD and quality of life were improved. As proposed in ERS/ESC guidelines, more data are required before appropriate recommendations can be made [15].

#### *Altitude and hypoxia*

As hypoxic vasoconstriction may be an aggravating factor in PAH, stays at altitude above 1500-2000 meters without supplemented oxygen and air flight in unpressurized cabin should be avoided [15]. Chronic hypoxia (PaO2 <60 mmHg) warrants oxygen therapy for symptoms and to avoid PAH deterioration.

#### *Pregnancy and contraception*

The hemodynamic and hormonal modifications occurring during pregnancy and peripartum period can lead to severe, and sometimes fatal, right heart failure [197,198]. Pregnancy is considered to be associated with high rate of mortality (30-50%) in PAH patients [15]. Thus, pregnancy is contraindicated in women affected by PAH. Consequently, contraception is strongly recommended in PAH women of childbearing age [197,198]. Combined estrogen-progestin oral contraceptive is theoretically contraindicated because their pro-thrombotic activity. Therefore, mechanical contraception (intra-uterine device for example) or surgical sterilization should be proposed. Nethertheless, pregnancy should be managed with specific PAH therapies and planned elective delivery in expert centres [15].

#### *Anaesthesia and surgery*

Hypotension induced by anaesthetic drugs, and hemodynamic insults following surgical and anaesthetic interventions are generally poorly tolerated by PAH patients, with a high procedural morbidity and mortality. Consequently, it is recommended in general that PAH patients avoid unnecessary procedures. When required, these should be highly planned with the multidisciplinary team, either within, or in close liaison with, the expert centers, in order to coordinate surgical and medical interventions [199].

#### *Proscribed drugs*

Vasoconstrictors used in cold medication to relieve nasal congestion should be avoided in PAH patients. Beta-blockers have been shown to be deleterious to PAH patients because they prevent the important adaptive physiologic response that allows preservation of adequate cardiac output. To discontinue such drugs in a patient with PAH may lead, by itself, to important clinical and hemodynamic improvement [200].

### Non specific drugs

#### *Diuretics*

Diuretics are one of the most important treatments in the setting of PAH because right heart failure leads to fluid retention, hepatic congestion, ascites and peripheral edema. Right ventricular overload is part of clinical symptoms and has been associated with a poor prognosis in PAH [201]. Diuretics and salt-free diet relieve hypervolemia and associated symptoms. Whether this strategy improves prognosis is unknown. Dose adjustment of diuretics is needed, based on clinical and hemodynamic findings. Renal function and blood chemistry should be monitored to avoid renal failure or dyskalemia [192].

#### *Oral anticoagulation*

Pathology specimens from PAH patients may show in situ thrombosis and thrombi recanalisation. Only few studies support anticoagulation treatment in PAH (mostly retrospective or not randomized) [202,203]. Current recommendations propose oral anticoagulation aiming for targeting an International Normalized Ratio (INR) between 1.5 and 2.5. Although the somewhat sparse evidence base is derived exclusively from idiopathic, heritable and PAH due to anorexigens, anticoagulation has been generalised to all patient groups, given the absence of contraindication. Anticoagulation is usually not recommended in porto-pulmonary hypertension because of the risk of esophageal variceal haemorrhage. In patients with systemic sclerosis, oral anticoagulation can be difficult to manage because of their high risk of bleeding, especially from the gastrointestinal tract. Variceal ligation is a preventive option in these cases. Long-term oral anticoagulation is essential in CTEPH with an INR which is recommended to be between 2 and 3.

#### *Digitalis*

Digoxin has been suggested as part of PAH therapy in the past because it produces an acute increase in cardiac output [204], although its efficacy is unknown in PAH. Therefore it is usually proposed in PAH associated with atrial tachyarrhythmias [192].

### Calcium channel blockers

Calcium channel blockers (CCB) are indicated in patients with a positive vasodilatation challenge test after inhaled NO. CCB are vasodilators and were initially introduced in the 1980’s as part of PAH therapy to counteract vasoconstriction that has traditionally been assumed to be a preponderant mechanism in PAH. In the 1990’s, Rich and colleagues showed in an open prospective study that high dose of calcium channel blockers (nifedipine 90 to 240 mg/day or diltiazem 360 to 900 mg/day) significantly improve prognosis in patients with an acute vasodilation response [203]. Sitbon et al. showed that only 12.6% out of the patients could be treated by CCB according to criteria's establish by Rich and co-workers [203]. Moreover, only half of them maintained actual long-term benefit defined as NYHA FC I or II at one year without the need for additional treatment [205]. In this study, a decrease in mean PAP > 10 mmHg to reach a value < 40 mmHg, with a stable or increased cardiac output, during acute vasodilator test was predictive of long-term response to CCB. Contrarily, CCB must be avoided in the absence of acute vasoreactivity because of the risk of significantly reduced cardiac output and systemic blood pressure [205]. The choice of CCB is based upon the patient’s heart rate; nifedipine and amlodipine were preferred in the presence of relative bradycardia and diltiazem in the presence of tachycardia. Doses of CCB used in this setting are relatively high, 120-240 mg de nifedipine, 240-270 mg for diltiazem, and up to 20 mg for amlodipine [15]. If the patient does not show a correct response (NYHA FC I or II with marked hemodynamic improvement), additional PAH specific therapy should be added. Of note, CCB is contraindicated in PVOD because of the high-risk of life threatening pulmonary edema, even in the presence of acute vasodilator response that can be observed in the same proportion as in idiopathic PAH [53].

### PAH-specific therapies

Better understanding in pathophysiological mechanisms of PH over the past quarter of a century has led to the development of medical therapeutics, even though no cure for pulmonary arterial hypertension exists. Several specific therapeutic agents were developed for the medical management of PAH including prostanoids, endothelin receptor antagonists and phosphodiesterase type 5 inhibitors. Furthermore, emerging treatments such as tyrosine kinase inhibitors, soluble guanylate cyclase activators (riociguat) and prostacyclin receptor agonists (selexipag) are currently being evaluated in PAH.

#### *Prostanoids*

Endothelium-derived prostaglandin I2 (PGI2), or prostacyclin, is an arachidonic acid produced by endothelial cells. Prostacyclin is a powerful systemic and pulmonary vasodilator and an inhibitor of platelet aggregation through the increase in intracellular cyclic adenosine monophosphate (cAMP) [206,207]. Moreover, prostacyclin plays an important role in antiproliferative, antithrombotic, antimitogenic and immunomodulatory activity [208]. Prostanoids are a family of prostacyclin analogues available in intravenous, subcutaneous, or inhaled form.

**Epoprostenol** In the 1980s, intravenous epoprostenol was the first prostanoid evaluated in PAH [209]. As the half-life of epoprostenol is <5 minutes, it requires an indwelling central venous catheter which is connected to an infusion pump for continuous intravenous administration. Treatment with epoprostenol is complex, uncomfortable and expensive and cannot be considered as an ideal treatment despite its evident clinical benefit. Common side effects associated with treatment include headache, flushing, jaw pain and gastrointestinal disturbance [201,210]. The efficacy of continuous i.v. administration of epoprostenol has been tested in three unblinded RCTs in idiopathic PAH [207,211] and PAH associated with systemic sclerosis [212].

Barst and colleagues [183] showed improvement in exercise capacity with an increase in 6-MWD of 47meters after 12 months of epoprostenol treatment in 81 patients with idiopathic PAH. Moreover, a significant improvement of survival was observed (no death at three months of treatment in the group with epoprostenol against 8 deaths in the group receiving conventional treatment including diuretics and anticoagulants [213]. Long-term persistence of efficacy has also been shown [201,214] in idiopathic PAH, as well as in other associated PAH [215,216] and in non operable CTEPH [217]. Long term effectiveness has never been evaluated prospectively but retrospective analysis comparing patients treated by intravenous epoprostenol with data from patients treated with conventional therapy find meaningful clinical benefit for patients in NYHA FC III or IV [201,214,218,219]. Functional class, hemodynamic parameters and long term survival were all improved in the group treated with i.v. epoprostenol.

Epoprostenol had initially been proposed as a bridging therapy for lung transplantation but it is currently regarded as the treatment of choice for patients in NYHA FC IV. If treatment with epoprostenol is necessary, it will be started at low dose of 2-4 ng/kg/min and is gradually increased to 10-16 ng/kg/min according to side effects [201]. Treatment interruption secondary to pump dysfunction or the rupture of the catheter, although rare, can induce serious adverse events [201]. Because of its route of administration, central venous catheter bloodstream infections can occur, and should be systematic searched in the context of unexplained clinical deterioration.

**Treprostinil** Due to complications related to the central venous catheter used for the i.v. administration of epoprostenol, other prostacyclin agonists have been developed. Treprostinil is a prostacyclin analogue which benefits from a longer half-life of 58–83 minutes by subcutaneous administration [220]. It is delivered by a minipump similar to those used for insulin [221]. A multicenter randomized trial evaluated subcutaneous administrated treprostinil versus placebo over three months in patients in NYHA functional class II-IV suffering from idiopathic PAH, PAH related to CHD with a shunt or PAH associated with CTD [221]. Patients treated with treprostinil increased 6-MWD and had benefits like quality of life, pulmonary hemodynamics and improvement of clinical symptoms. Unfortunately, local side-effects such as pain and inflammation at the injection site are present in the majority of patients treated with treprostinil which often lead to limitation of increasing dose or treatment cessation. Intravenous treprostinil is only licensed for the use in the USA and provides the advantage of less frequent need for drug reservoir replacement [220]. Inhaled treprostinil was examined in a placebo-controlled TRIUMPH-1 study [222], but results of this formulation were not convincing and this product is not yet licensed outside the USA.

**Inhaled Iloprost** Iloprost is a prostacyclin analogue administered by inhalation or the intravenous route. The pulmonary vasodilating effects of inhaled iloprost lasts nearly 45 minutes, therefore six to nine inhalations daily are needed, with each of them requiring approximately 30 minutes [223,224]. Common side effects of treatment were cough, flushing, jaw pain and headache [223]. In the AIR study [223] conducted in at 37 European pulmonary hypertension centers, study participants (idiopathic PAH, PAH associated to systemic sclerosis, anorexigen associated PAH or non operable CTEPH) in functional class III or IV were assessed during a three month period. Patients received a daily inhalation of 2.5 μg or 5.0 μg of iloprost 6 or 9 times a day or placebo. After 3 months of treatment, 17% of patients receiving iloprost reached the combined endpoint of improvement in functional class and 10% gain in 6-MWD as compared to 5% in the placebo group.

#### *Endothelin receptors antagonists*

Endothelin-1 (ET-1) is a potent vasoconstrictor and therefore plays an important role in the pathogenesis of PAH. In addition, it is responsible for smooth muscle cell proliferation [158]. Elevated ET-1 plasma levels are found in patients suffering from PAH and are correlated with poor prognosis. There are two existing isoforms of ET-1 receptors: endothelin A (ETRA) and endothelin B (ETRB). Activation of ETRA and ETRB on pulmonary artery smooth muscle cells induce proliferation and vasoconstriction, whereas activation of ETRB on pulmonary endothelial cells leads to release of NO and prostacyclin and participate to the clearance of circulating ET-1.

**Bosentan** Bosentan is an oral active dual ETRA and ETRB antagonist. Bosentan has been evaluated in PAH (idiopathic, associated with CTD, and Eisenmenger’s syndrome) in five RCT’s (Pilot, BREATHE-1, BREATHE-2, BREATHE-5, and EARLY) that have shown improvement in exercise capacity, functional class, haemodynamic, echocardiographic and Doppler variables, and time to clinical worsening [107,225-228]. The first placebo-controlled study included 32 patients affected by idiopathic PAH or scleroderma presenting with PAH showing significant exercise improvement with a gain of 76 meters after three months of treatment with bosentan as compared to placebo [225].

The BREATHE 1 study confirmed the efficacity of treatment with Bosentan in more than 200 assessed patients in NYHA functional class III or IV compared to placebo in a three month trial. After three months of treatment with bosentan, improvements in NYHA functional class were observed in 42% of patients receiving bosentan compared with 30% in the placebo arm. 6-MWD was improved overall to 44 meters in favor of bosentan. Furthermore, delayed time to clinical worsening was also noted as well as better results in dynpnea scores [226]. One accepted common side effect of treatment is increase in liver enzymes; this is why monthly monitoring of liver transaminases is mandatory in all patients receiving bosentan. Treatment is started at a dose of 62.5 mg twice a day and increased to the dose of 125 mg twice daily after one month of treatment.

Subsequently published data of treatment with bosentan suggested persistent improvements in pulmonary hemodynamics, exercise capacity and modified NYHA functional class and possibly survival rate of patients [229-231]. Results from the EARLY study (double-blind, randomised controlled 6 months trial) showed that the effect of the dual endothelin receptor antagonist bosentan in patients with mildly symptomatic PAH could be beneficial for PAH patients in NYHA functional class II [228].

**Ambrisentan** Ambrisentan is a selective ETA receptor antagonist administrated once daily at a dose of 5 mg or 10 mg. Two large RCTs (ARIES I and II, i.e. Ambrisentan in Pulmonary Hypertension, Randomized, Double blind, Placebo-controlled Multicenter, Efficacy Studies I and II) have demonstrated efficacy on symptoms, haemodynamics and time to clinical worsening of patients with idiopathic PAH and associated to CTD and HIV infection [232]. An extension study of the ARIES study is the recently published ARIES-E study by Klinger and colleagues [233]. They followed patients for a mean period of 60 weeks in where patients underwent hemodynamic evaluation. The authors concluded that treatment with ambrisentan leads to hemodynamic stability in PAH patients.

#### *Phosphodiesterase type-5 inhibitors*

NO is a potent pulmonary arteries SMC relaxant that disposed vasodilator activity through up-regulation of its associated down-stream signalling molecule, cyclic GMP (cGMP), metabolism of which is dependent on the activation of a number of PDEs [208]. Phosphodiesterase type 3, 4 and 5 are the three main types of this enzyme found in pulmonary artery contractive cells. PDE-5 is the most abundantly expressed isoform in pulmonary circulation which was confirmed by several experimental investigations showing a beneficial effect of PDE-5 inhibitors on vascular remodelling and vasodilatation [234,235].

**Sildenafil** Sildenafil is an oral PDE-5 inhibitor that is available in Europe since 2005 for PAH patients in functional class II-III whereas this drug is licensed in Canada and the USA for patients in functional class II-IV. Basis for the authorization of this drug in the setting of PAH was a large randomized, placebo-controlled trial in which different doses of sildenafil were assessed in 278 patients presenting with idiopathic PAH, PAH related to CTD or congenital systemic to pulmonary shunts surgically corrected. The majority of study patients were in functional class II-III. After three months of treatment, the mean placebo-adjusted changes in 6-MWD for 20 mg, 40 mg and 80 mg doses of sildenafil were 45 meters, 46 meters and 50 meters, respectively. Furthermore, significant hemodynamic and functional class improvements were noted in every sildenafil group as compared to placebo. Common side effects of treatment with sildenafil include headache, flushing and dyspepsia but no hepatic enzymes increase was noted as compared with endothelin receptor antagonists. Long term extension data from 222 patients who completed one year of sildenafil monotherapy with a dose of 80 mg three times daily showed encouraging results with a gain in 6-MWD, suggesting a durable treatment effect [236]. However, sildenafil approval in Europe is currently limited to 20 mg three times a day. No data is currently available on the long-term efficacy of this lower dosage.

**Tadalafil** Another PDE-5 inhibitor is tadalafil which was granted for use in patients with PAH in Europe and North America in 2009. Galiè and colleagues [237] assessed in the PHIRST trial 405 randomly assigned patients who were either treatment naïve or already receiving bosentan therapy to placebo or one of the several proposed doses of tadalafil 2.5 mg or 10 mg or 20 mg or 40 mg once daily for a period of three months. At study completion, patients receiving tadalafil showed an overall mean placebo-correlated increase in 6-MWD of 33 meters [208]. Thus, this increase was dose-dependent, with only the 40 mg dose achieving the prespecified value for statistical significance for improvement. Data analysis of comparative hemodynamic data from 93 patients who underwent repeat RHC has significant reduction in mPAP and PVR under treatment with tadalafil. Barst and co-authors [238] underlined favourable effects with tadalafil 40 mg among patients receiving background bosentan, although improvements were less marked compared with treatment naïve cohort.

### Combination therapy

Another therapeutic option is to combine drugs with different mechanisms of action, in order to optimize clinical benefit while minimizing side effects. The two possible strategies for combination therapy are either to add a new medication to an ongoing treatment (sequential combination) or in first intention by starting from the beginning with a combination treatment. The BREATHE-2 trial compared the association of i.v. epoprostenol and oral bosentan with i.v. epoprostenol and placebo among 33 patients with severe PAH over a 12 weeks period [227]. Reduction in pulmonary vascular resistance was greater with combination therapy, although it did not reached statistical significance. Furthermore, no benefit could be shown on the 6-MWD with combination therapy compared to epoprostenol alone (+ 68 m and + 74 m, respectively). These results however may be related to the small number of patients and the short term follow up in the context of the addition of a treatment to a drug already known to bring an important benefit in severe PAH patients. In a recent controlled trial, the sequential addition of oral sildenafil 80 mg three times daily for patients already receiving i.v. epoprostenol with insufficient improvement, proved to be more effective than the placebo on the 6-MWD and hemodynamic parameters. Indeed, there was a significant reduction in the number of patients showing clinical worsening and an improvement of survival among most severe patients [239]. There is currently no data on the addition of bosentan in such context. The combination of iloprost to bosentan in patients with idiopathic PAH or associated PAH in NYHA functional class III was shown to be significantly better than placebo and bosentan in terms of 6-MWD (+ 26 m), NYHA functional class and on post inhalation hemodynamic parameters (PVR −26.4%) [240].

Several uncontrolled studies evaluated the efficacy of other associations with encouraging results. The open-label sequential addition of bosentan or sildenafil to epoprostenol, treprostinil or iloprost was shown to be of interest [241-243]. Also, the association of ERA and PDE5, both available in oral form, offers an interesting option [244,245]. Currently, the limited data precludes consensus on which combined treatment or strategy should be preferred. Also, no long term evaluation of combination therapy is available.

The best strategy of combination therapy should be discussed in the next world symposium of Pulmonary Hypertension. Current ERS/ESC guidelines propose sequential combination therapy without any recommendation regarding the best way to associate available specific PAH therapies.

### Potential future therapies

#### *Macitentan*

Macitentan, also called ACT-064992, is a novel, highly potent, tissue-targeting dual ET-1 receptor antagonist characterized by a high lipophilicity [247]. Macitentan has been tested in the largest, long-term, event-driven randomized, controlled study (SERAPHIN, *Study with an Endothelin Receptor Antagonist in Pulmonary arterial Hypertension to Improve clinical outcome*) [246]. The Seraphin study was designed to evaluate the efficacy and safety of macitentan through the primary endpoint of time to first morbidity and all-cause mortality event in 742 patients with symptomatic PAH. Patients were treated for up to three and a half years.

Macitentan has met its primary endpoint, decreasing the risk of a morbidity/mortality event over the treatment period versus placebo. Secondary efficacy endpoints, including change from baseline to month six in 6-MWD, change from baseline to month six in NYHA FC and time – over the whole treatment period - to either death due to PAH or hospitalization due to PAH, also showed a dose-dependent effect. Treatment with macitentan in this study was well tolerated; headache, nausea and vomiting were reported as minor adverse events [248]. The safety set comprised 741 patients, who received at least one dose of study treatment (placebo, 3 mg or 10 mg). The number of adverse events reported and patients discontinuing treatment due to adverse events was similar across all groups. Similar elevations of liver aminotransferases greater than three times the upper limit of normal were observed in all groups. In addition, no difference was observed between macitentan and placebo in terms of fluid retention (edema). A decrease in hemoglobin - reported as an adverse event - was observed more frequently on macitentan than placebo, with no difference in treatment discontinuation between groups.

#### *Vardenafil*

Vardenafil is another PDE5 inhibitor. Recently published data from a prospective, randomised study including 66 patients with PAH suggests improvement in 6-MWD and hemodynamic parameters after 3 months of treatment with vardenafil as compared to placebo. Side effects are not reported and proposed dosage is 5 mg twice daily [249]. However, further studies are needed.

#### *Riociguat*

Endothelium-derived NO regulates vascular homeostasis through pulmonary arteries SMC relaxation *via* the activation of the second messenger cGMP. The clinical benefits associated with the PDE-5 inhibitor class has led to interest in testing whether other agents that modulate NO signaling might be similarly beneficial in PAH. Riociguat is a first-in-class drug that augments cGMP biosynthesis through direct stimulation of the enzyme soluble guanylate cyclase (sGC) promoting vasodilatation by direct stimulation of sGC in an NO-independent fashion, and by sensitization of sGC to low endogenous NO levels [250].

A phase I randomized placebo-controlled study in 58 healthy male subjects were given riociguat orally was designed to test the safety profile, pharmacokinetics and pharmacodynamics of single oral doses of riociguat (0.25–5 mg). A proof-of-concept study was conducted to investigate oral riociguat in patients with moderate to severe PH in a two-part, non-randomized, open-label, single center trial [251]. Riociguat was well tolerated in doses up to 2.5 mg, whereas 5 mg caused asymptomatic hypotension in one patient. Therefore the 2.5 mg dose was used in the second part of the trial to demonstrate efficacy. Riociguat significantly reduced mPAP, PVR and systemic vascular resistance and increased cardiac index [251]. Results from a multicenter, open-label, uncontrolled phase II trial involving 75 patients with PAH (n = 33) and chronic thrombo-embolic PH (n = 42) showed that 12 weeks of oral riociguat given 3 times daily conferred improvements in symptoms, NYHA FC, exercise capacity, NT-proBNP level, and pulmonary hemodynamics [252]. Riociguat is also under investigation in other form of PH as PH associated with chronic obstructive pulmonary disease, with interstitial lung disease or with left ventricular dysfunction [253-255].

The Phase III, double-blind, randomized, placebo-controlled PATENT-1 study investigated the efficacy and safety of riociguat in patients with symptomatic PAH [256]. Treatment-naïve patients and patients pre-treated with ERAs or prostacyclin analogues were eligible. Riociguat was titrated from a starting dose of 1 mg three times daily (t.i.d.) [range 0.5–2.5 mg t.i.d.]. The primary outcome was the change from baseline in 6-MWD at week 12. Secondary endpoints included the change from baseline in pulmonary vascular resistance (PVR), NT-proBNP, NYHA FC, clinical worsening, living with PH questionnaire and Borg dyspnea score. A total of 445 patients were randomized. Preliminary analysis showed a significant increase in 6-MWD from baseline of 35.8 m with riociguat versus placebo (95% CI 20.1–51.5 m, p < 0.0001). Predefined exploratory analyses indicated that riociguat improved 6-MWD in pretreated patients (+35.7 m [95% CI 15.0–56.3 m]) as well as treatment naïve patients (+38.4 m [95% CI 14.5–62.3 m]). Significant improvements were also seen in PVR (p < 0.0001), NT-proBNP (p < 0.0001), functional class (p = 0.003), clinical worsening (p = 0.0046), living with PH questionnaire (p = 0.002) and Borg dyspnea score (p = 0.002). Riociguat was well tolerated and had a favorable safety profile. Thus, riociguat may represent a new treatment option for patients with PAH. An open-label extension study (PATENT-2) will evaluate the long-term safety of riociguat in patients with PAH.

#### *Selexipag*

Selexipag is an orally active prodrug metabolized to the highly selective prostacyclin IP receptor agonist ACT-333679 (previously known as MRE-269), which has a half-life of over 6 h [257]. With in high selectivity for the IP receptor over other prostanoid receptors (at least 130-fold selectivity), selexipag can be distinguished from beraprost or iloprost currently used in the management of PAH [258]. With no affinity for the prostaglandin E receptor 3 (EP3), selexipag exerts similar vasodilatory activity on both large and small pulmonary arterial branches [259] and its relaxant efficacy is not modified under conditions associated with PAH, whereas relaxation to treprostinil may be limited in the presence of mediators of disease such as ET-1 [260]. Preclinical study results showed that twice-daily administration of selexipag attenuates right ventricular hypertrophy, improves pulmonary hemodynamics, and significantly increases survival in MCT-treated PH rats [259].

A phase II study, involving 43 PAH patients showed that treatment with selexipag for 17 weeks conferred significant improvements in PVR (−30.3% versus placebo) compared with placebo [261]. Treatment with selexipag was well tolerated by most patients in this study. Adverse events were consistent with the known side effect profile of IPr agonism such as headache, pain in extremity, pain in jaw, nausea, and diarrhea. These side effects decreased over time in patients treated with selexipag [261].

A phase III randomized trial GRIPHON [262] to examine the effect of selexipag on morbidity and mortality in PAH is underway and will afford more information regarding efficacy and safety of selexipag.

#### *Tyrosine kinase inhibitors*

One of the most promising targets in PAH is platelet-derived growth factor (PDGF). PDGF has been implicated in endothelial cell dysfunction and proliferation and migration of smooth muscle cells. It has been suggested that PDGF may play a role in PAH [263-265]. Pulmonary vascular remodelling in different animal models of PAH was shown to regress with the administration of imatinib mesylate (Gleevec®), a PDGF receptors antagonist approved for the treatment of chronic myeloid leukaemia [266]. Moreover, case reports suggest a beneficial effect of imatinib among severe PAH patients and a first randomized (imatinib vs placebo), double-blind, 24 weeks Phase II study was performed in 59 PAH patients in NYHA functional class II to IV receiving specific PAH therapies [267]. The primary endpoint (6-MWD after 24 weeks) was not different between 2 groups (+22 m in imatinib group as compared to placebo) even if a significant improvement of hemodynamic parameters was observed, especially among the patients with the most severe hemodynamic compromise [267]. This preliminary study does not allow concluding on the potential benefits of imatinib in PAH, but led to development of a phase III clinical trial (IMPRES) evaluating imatinib in a randomized controlled double-blind trial of 24-week, in 202 severe PAH patients treated with at least two PAH specific drugs. After 24 weeks, a significant improvement in the primary endpoint, 6MWD, was observed (+32 m in imatinib group vs. placebo) as well as an improvement of secondary endpoints including hemodynamic parameters [268]. However, several cases of subdural hematoma were reported in patients treated with imatinib: 2 cases in the double-blind period of 24 weeks and 7 supplemental cases in an open-label extension phase of the study. The mechanism of these subdural hematomas is not elucidated and the high incidence observed may be partly favored by anticoagulation recommended for PAH patients. Subdural hematoma is a complication of imatinib previously reported in other settings where it has been used, including oncology or pulmonary fibrosis. Netherless, this complication has rarely been reported in previous clinical trials in PAH or in registries. In addition, it has been demonstrated that tyrosine kinase inhibitors, including imatinib, may have possible cardiac adverse effects on long term use that might limit its benefice in PAH [269]. According to these results, the benefit/risk ratio of imatinib in PAH was not considered to be sufficient and to date, the use imatinib was not recommended in PAH.

### Non medical treatment

#### *Balloon atrial septostomy*

The presence of a right-left shunt, secondary to congenital cardiac malformation, or a patent foramen oval, among patients with severe PAH, seems to carry a better prognosis [270]. Atrioseptostomy is an artificial communication interauricular to decrease the right heart volume, subjected to a high after load secondary to increased pulmonary resistances [271]. The surgical creation of a right-left shunt decreases right auricular pressure and increases systemic blood flow, later on, reduction in right ventricular wall tension is expected [271]. Thus, in spite of arterial desaturation induced by the shunt, oxygen delivery is improved [271]. Atrioseptostomy has however never been studied in controlled clinical trials. However, several experienced teams reported their data and it seems that immediate mortality is high, reaching 14% during the first week, particularly in the case of severe desaturation and right heart failure [105,271-273]. Among patients who survived, clinical improvement with regression of symptoms and gain of functional capacity can be observed. Atrioseptostomy should only be carried out in centers with significant experience, both in performance of atrioseptostomy and management of PAH patients, especially post interventional. The impact of balloon atrial septostomy on long term survival has not been established in RCTs [271,274].

#### *Lung transplantation*

Lung transplantation was historically the treatment of choice for severe PAH and remains treatment of choice if medical treatments are insufficient [197]. However, this particularly heavy surgery can be proposed only to a minority of patients suffering from PAH. Moreover, long-term benefits remain disappointing with approximately 50% survival at 5 years [275]. Early mortality is mainly related to infectious complications whereas late mortality reflects mostly chronic rejection such as obliterating bronchiolitis. Mono-pulmonary transplantation had good long-term results [276,277], but most centers currently prefer bi-pulmonary transplantation which has less post-operative complications [278]. Cardiopulmonary transplantation may be necessary for patients presenting with terminal right heart failure or complex congenital heart disease [279]. In conclusion, PAh is a rare group of diseases that shares broadly similar pathological features, pathophysiology and clincal presentation. The discovery of the central role of endothelial dysfunction leads to the development of specific PAH therapies including prostanoids, endothelin receptor antagonists and PDE-5 inhibitors. Even these therapeutic advances, no cure of the disease can be achieved and the prognosis remains unsatisfactory.

## Abbreviations

ALK-1: Activin A receptor type II-like kinase-1; BMPR2: Bone morphogenetic protein receptor 2; CCB: Calcium channel blockers; CHD: Congenital heart diseases; Chest HRCT: Chest High resolution computed tomography; CI: Cardiac index; CMR: Cardiac magnetic resonance imaging; CO: Cardiac output; CTD: Connective tissue disease; CPET: Cardiopulmonary exercise testing; CTEPH: Chronic thrombo-embolic pulmonary hypertension; DLCO: Diffusing lung capacity of carbon monoxide; DLCO/VA: Diffusing lung capacity of carbon monoxide/alveolar volume; ESC: European Society of Cardiology; ERS: European Respiratory Society; HHC: Hereditary hemorrhagic telangiectasia; HIV: Human immunodeficiency virus; INR: International Normalized Ratio; NIH: National Institute of Health; NO: Nitric oxide; NYHA: New York Heart Association; PAH: Pulmonary arterial hypertension; PFT: Pulmonary function test; mPAP: Mean pulmonary arterial pressure; PCWP: Pulmonary capillary wedge pressure; PVOD: Pulmonary veno-occlusive disease; PVR: Pulmonary Vascular Resistance; RAP: Right atrial pressure; PaO2: Partial pressure of arterial oxygen; PaCO2: Partial pressure of arterial carbon dioxide; PDGF: Platelet-Derived Growth Factor; RCT: Randomized clinical trial; RHC: Right heart catheterization; SpO2: Pulse arterial oxygen saturation; SR: Sex ratio; SvO2: Mixed venous oxygen saturation; TTE: Transthoracic Doppler-echocardiography; TKI: tyrosine kinsase inhibitor; TLC: Total lung capacity; TPR: Total Pulmonary Resistance; TGF: beta Transforming growth factor beta; VEGF: Vascular endothelial growth factor; 6-MWD: 6-minute walk distance.

## Competing interests

D Montani, X Jaïs, L Savale, M Humbert, G Simonneau and O Sitbon have been supported by Actelion, Bayer Schering, GlaxoSmithKline, Novartis, Pfizer, Inc., Lilly & Co., and United Therapeutics for consultancy services and as members of their scientific advisory boards. The other authors declare that they have no competing interests.

## Authors’ contributions

DM, SG, PD, FP, BG, GG, XJ, LS, EAM, LCP, MH, GS, OS participated in drafting the manuscript. All authors read and approved the final manuscript.
